# Paliperidone Palmitate Associated with Necrotizing Deep Tissue Infection and Sepsis Requiring Surgical Intervention

**DOI:** 10.1155/2015/364325

**Published:** 2015-12-30

**Authors:** Jonathan G. Leung, Kirstin J. Kooda, Erin N. Frazee, Sarah Nelson, Katherine M. Moore

**Affiliations:** ^1^Department of Pharmacy, Mayo Clinic, Rochester, MN 55902, USA; ^2^Department of Psychiatry and Psychology, Mayo Clinic, Rochester, MN 55902, USA

## Abstract

Long-acting injectable antipsychotics provide the delivery of medication over an extended period of time requiring administration typically only every 2 to 4 weeks. The side effect profile of a long-acting injectable antipsychotic is predictable and similar to the oral formulation. However, injection site reactions may occur with this novel delivery system. The risk of an injection site reaction may be greater with the repeated administration of a lipophilic decanoate formulation and include pain, development of indurations, and fibrosis. Severe complications from injection site reactions have rarely been described in the literature with newer agents. We report the first case of a patient prescribed paliperidone palmitate every 3 weeks that developed severe sepsis requiring vasopressors and intubation due to delayed relayed recognition of a necrotizing infection at an injection site. Clinicians should be alerted to screen for injection site reactions when there is an unknown source infection in a patient receiving a long-acting injectable antipsychotic.

## 1. Introduction

Long-acting intramuscular antipsychotics (LAIAs) are often utilized in patients with schizophrenia and have a number of clinical advantages related to medication adherence. A single administered dose of an LAIA can provide constant delivery of mediation over multiple weeks. The LAIA, monthly paliperidone palmitate, can be detected in the serum for up to 6 months after discontinuation and over 1 year with the every 3-month formulation [1, 2]. Thus acknowledgement that a hospitalized patient had received an LAIA prior to admission is crucial when evaluating for drug-interactions and adverse events. The side effect profile for oral antipsychotic medications is similar to the same medication in an LAIA dosage form. However, specific to an LAIA formulation is the risk for injection site reactions. The risk for injection site reactions may be influenced by multiple factors such as dose, drug concentration, and whether the drug delivery vehicle is lipophilic or hydrophilic [3, 4]. For paliperidone palmitate injection site reactions were reported in clinical trial as infrequent, mild, decreasing over time, and with similar incidence compared to placebo [1]. We describe the case of a patient prescribed paliperidone palmitate that developed severe sepsis, requiring vasopressors, and mechanical ventilation, due to delayed recognition of a necrotizing infection at the injection site.

## 2. Case Presentation

A 26-year-old female weighing 77 kg with a past medical history of schizophrenia presented to an outside emergency department (ED) with a one-week history of chills, dizziness, and diffuse myalgias. As an outpatient she was prescribed paliperidone 1.5 mg by mouth daily and paliperidone palmitate 234 mg intramuscular (IM) every 3 weeks. Her last intramuscular paliperidone palmitate injection was 11 days prior to presentation at the outside ED and given in her right deltoid. The dosing interval was shortened from every 4-week injection, 3 months prior to this hospital admission. This was due to breakthrough symptoms that occurred at the end of every 4-week frequency. Outpatient records indicated that the plan was to inevitably discontinue the oral paliperidone. Records also indicated that the paliperidone palmitate was initiated 14 months prior to presentation.

Following evaluation in the ED, the patient was transferred to the medical intensive care unit due to tachycardia, hypotension, fever, tachypnea, leukocytosis (37,400/mm^3^), and metabolic derangements (i.e., serum values: potassium 2.2 mEq/mL, magnesium 0.9 mg/dL, lactate 6.9 mmol/L, and chloride 113 mmol/L; arterial blood gases: PO2 67.4 mmHg, PCO2 26.4 mmHg, pH 7.2). She was given 7 liters of intravenous (IV) fluids, while she was also initiated on piperacillin/tazobactam and vancomycin. Medically, the patient's presentation and laboratory findings were concerning for septic shock of unclear etiology. A urinalysis was negative and there were no acute findings on computed tomography with IV contrast of the chest, abdomen, and pelvis. An abdominal ultrasound of the gallbladder did not reveal significant findings. Over the course of the first two ICU days, the patient's condition deteriorated and she required intubation and high-dose vasopressors, 0.84 mcg/kg/min of norepinephrine, 0.04 units/min of vasopressin, and phenylephrine 3 mcg/kg/min. Fevers greater than 39°C, leukocytosis (37,400/mm^3^), and hemodynamic instability persisted into hospital day 2. Blood cultures at this time exhibited no growth and a bronchoalveolar lavage was unrevealing for infection, but levofloxacin was added empirically to the antibiotic regimen given the patient's worsening condition. Psychiatry consultation noted features similar to neuroleptic malignant syndrome (NMS). However, on prior and current physical exams muscle stiffness or rigidity was not present making NMS a less likely explanation for her current condition. By day 6 of hospitalization, the patient's hemodynamics stabilized allowing for discontinuation of vasopressors; but respiratory failure, fever, and leukocytosis persisted.

**Figure 1:**
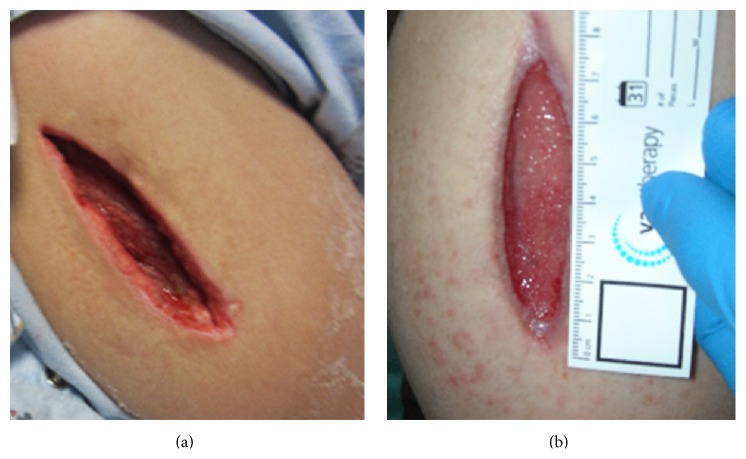
Right upper extremity 10 (a) and 54 (b) day after surgical debridement.

With the lack of objective evidence on imaging and microbiology, noninfectious sources of fever and leukocytosis were considered. Also antibiotics were discontinued to rule out drug-fever but the patient's temperature remained elevated. Lower extremity ultrasounds were negative bilaterally for thromboses and hepatology and rheumatology consults did not elicit any additional etiologies for the ongoing symptomatology. On hospital day 10, indium leukocyte imaging found a probable infection of the right shoulder and right anterolateral distal lower extremity. It was near this time that the patient was also given 200 mg of methylprednisolone which correlated to a decrease of body temperature and improvement of leukocytosis. Given temporal response to a corticosteroid, polymyositis or similar inflammatory myositis was considered and a muscle biopsy was scheduled. However, upon attempt to biopsy the right upper extremity, frank pus was noted emerging from the biopsy site. In an emergent and unplanned operative intervention, 250 mL of purulent fluid was drained from a necrotizing soft tissue infection. Vancomycin, meropenem, and clindamycin were started immediately after procedure, and the microbiology from the shoulder exhibited a positive stain for Gram-positive cocci resembling* Staphylococcus*. The next day the patient returned to the operating room for additional irrigation and debridement of the shoulder area, followed by placement of a wound vacuum assisted closure device. The patient was then extubated and transferred out of the ICU where she completed a 10-day course of daptomycin from the time of source control. She was discharged after a hospital stay of 26 days with additional wound care follow-up needed (Figure 1).

## 3. Discussion

The use of second-generation LAIAs has increased compared to first-generation LAIAs because they are associated with a decreased risk of extrapyramidal symptoms and NMS [5]. Second-generation LAIAs, including paliperidone palmitate, olanzapine pamoate, risperidone, and aripiprazole monohydrate, are also reported to have limited, mild injection site reactions (with the exception of olanzapine pamoate and the associated postinjection delirium/sedation syndrome). The second-generation LAIAs are all in various types of hydrophilic vehicles which may account for the lower incidence of injection site reactions compared to older agents. A review of literature and discussion with the manufacturer revealed no cases of abscess formation, necrosis, or sepsis associated with paliperidone palmitate. It should be noted, however, that there are no cases in the literature describing severe injection site reactions with risperidone LAIA but the package insert describes postmarketing surveillance reports of abscess, cellulitis, cyst, hematoma, necrosis, and isolated cases of required surgical intervention [6]. Thus it is possible that with more clinical experience more severe injection site reactions may be reported with paliperidone palmitate. Limited studies have been published describing the injection site reactions with the first-generation LAIAs. The FDA approved first-generation LAIAs include haloperidol decanoate and fluphenazine decanoate which are encompassed in a sesame oil vehicle (i.e., lipophilic). The prevalence of injection site reactions with the decanoate agents has been reported to be nearly 20% over a 12-month time period [4, 7]. Types of reactions described have included pain, lumps, indurations, and formation of scar tissue over time.

Our patient's clinical course was complicated by an unrecognized infectious process in the deltoid where the LAIA paliperidone palmitate had been administered. It was later confirmed, after the patient was extubated, that she had been experiencing increasing right shoulder pain starting 2 days after her most recent injection. This delayed identification of the infectious process was associated with an increase of ICU resource utilization, including increased length of stay and time on the ventilator. It was not until nearly 2 weeks into admission that there was an inadvertent discovery of abscess and necrotic tissue. It is not possible to know the exact mechanism in which the shoulder infection began but with all injection site reactions administration technique and sterile processes should be questioned. We also could not investigate the product for contamination as it administered prior to admission. It is possible that the patient's dosing had increased the risk for this injection site adverse event. The patient's dose was 234 mg every three weeks and above the FDA approved maximum dosing of 234 mg every 4 weeks. The high dose and more frequent administration may be a risk factor for injection site reactions [4]. The clinical rationale for the dosing was clear; however, in that every 4-week administration of paliperidone palmitate resulted in the emergence of psychotic symptoms 3 weeks after an injection. When the dosing interval was changed, the patient was reported to have improved control of psychotic symptoms.

Interestingly, we noted a high vasopressor requirement during the patient's early presentation of sepsis. While the requirement of multiple vasopressors is not uncommon in septic shock, the patient's antipsychotic use may have played a role in the need for high dose vasopressors. Several SGAs cause clinically significant dose-related peripheral blockade of alpha-1 adrenergic receptors in addition to their primary action of D2 receptor antagonism in the central nervous system. Paliperidone palmitate has moderate alpha-1 adrenergic activity and it has been previously reported that antipsychotics with alpha-1 adrenergic receptor antagonism can alter the effectiveness of vasopressors [8, 9].

We report a case in which a patient receiving paliperidone palmitate, an LAIA, experienced septic shock related to an unidentified abscess associated with the injection site of the medication. This case reminds clinicians that injection site reactions are possible with all LAIAs and that injection site infection should be ruled out when a patient presents with sepsis. Due to the limited data published on injection site reactions, clinicians should continue to report such events. Further study is required to assess if antipsychotics with alpha-adrenergic blockade affect vasopressor requirements in the setting of severe sepsis.
